# Static and Dynamic Cross‐Network Functional Connectivity Shows Elevated Entropy in Schizophrenia Patients

**DOI:** 10.1002/hbm.70134

**Published:** 2025-02-09

**Authors:** Natalia Maksymchuk, Robyn L. Miller, Juan R. Bustillo, Judith M. Ford, Daniel H. Mathalon, Adrian Preda, Godfrey D. Pearlson, Vince D. Calhoun

**Affiliations:** ^1^ Tri‐Institutional Center for Translational Research in Neuroimaging and Data Science (TReNDS): Georgia State University Georgia Institute of Technology and Emory University Atlanta Georgia USA; ^2^ Department of Psychiatry and Behavioral Sciences University of New Mexico Albuquerque New Mexico USA; ^3^ Department of Psychiatry and Behavioral Sciences University of California San Francisco California USA; ^4^ Mental Health Service San Francisco Veterans Affairs Healthcare System San Francisco California USA; ^5^ Department of Psychiatry and Human Behavior University of California Irvine California USA; ^6^ Departments of Psychiatry and Neuroscience Yale University School of Medicine New Haven Connecticut USA; ^7^ Institute of Living Hartford Healthcare Corp Hartford Connecticut USA

**Keywords:** biomarkers, brain states, dynamic functional connectivity, entropy, fMRI, functional connectivity patterns, image data analysis, mental health, schizophrenia, static functional connectivity

## Abstract

Schizophrenia (SZ) patients exhibit abnormal static and dynamic functional connectivity across various brain domains. We present a novel approach based on static and dynamic inter‐network connectivity entropy (ICE), which represents the entropy of a given network's connectivity to all the other brain networks. This novel approach enables the investigation of how connectivity strength is heterogeneously distributed across available targets in both SZ patients and healthy controls. We analyzed fMRI data from 151 SZ patients and 160 demographically matched healthy controls (HC). Our assessment encompassed both static and dynamic ICE, revealing significant differences in the heterogeneity of connectivity levels across available functional brain networks between SZ patients and HC. These networks are associated with subcortical (SC), auditory (AUD), sensorimotor (SM), visual (VIS), cognitive control (CC), default mode network (DMN), and cerebellar (CB) functional brain domains. Elevated ICE observed in individuals with SZ suggests that patients exhibit significantly higher randomness in the distribution of time‐varying connectivity strength across functional regions from each source network, compared to HC. C‐means fuzzy clustering analysis of functional ICE correlation matrices revealed that SZ patients exhibit significantly higher occupancy weights in clusters with weak, low‐scale functional entropy correlation, while the control group shows greater occupancy weights in clusters with strong, large‐scale functional entropy correlation. K‐means clustering analysis on time‐indexed ICE vectors revealed that cluster with highest ICE have higher occupancy rates in SZ patients whereas clusters characterized by lowest ICE have larger occupancy rates for control group. Furthermore, our dynamic ICE approach revealed that in HC, the brain primarily communicates through complex, less structured connectivity patterns, with occasional transitions into more focused patterns. Individuals with SZ are significantly less likely to attain these more focused and structured transient connectivity patterns. The proposed ICE measure presents a novel framework for gaining deeper insight into mechanisms of healthy and diseased brain states and represents a useful step forward in developing advanced methods to help diagnose mental health conditions.

## Graphical abstract

Patients with schizophrenia exhibit elevated inter‐network connectivity entropy spanning a wide range of functional brain networks, indicating higher randomness in connectivity strength distribution across various functional brain regions.

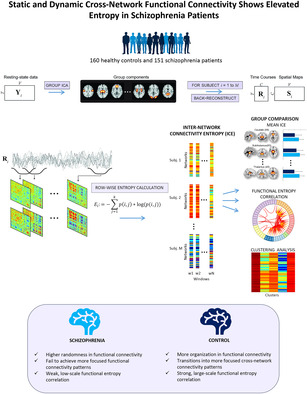

## 1 Introduction

Advancing tools designed to provide quantitative biomarkers for various psychiatric disorders is of increasing interest. Such tools seek to enhance the diagnostic accuracy and ability to screen for a given syndrome, while potentially offering further insights into the underlying neural mechanisms of mental disorders (Racz et al. 2020). Resting‐state (task‐free) functional magnetic resonance imaging (rs‐fMRI) is widely used for identifying characteristic and reproducible brain activation patterns associated with distinct cognitive and clinical conditions (Allen et al. 2014; Arbabshirani et al. 2013; Damaraju et al. 2014; Du et al. 2020; Li et al. 2020; Liu et al. 2008; Lurie et al. 2020; Miller et al. 2016a; Sakoğlu et al. 2010). It capitalizes on the relatively high metabolic rate of the brain, even at rest (Raichle and Gusnard 2002). In contrast to task‐based fMRI, rs‐fMRI is obtained without structured stimuli or tasks, allowing for the capture of the brain's spontaneous activity during rest. Thus, rs‐fMRI allows to explore spatiotemporal organization of the brain on macro‐scale level. The primary signal utilized in rs‐fMRI is the blood oxygenation‐level dependent (BOLD) data, reflecting alterations in oxygenation levels associated with neural activity across various brain regions. From a clinical perspective, rs‐fMRI provides several advantages. It is a non‐invasive and relatively straightforward‐to‐administer technique with fewer demands on subjects compared to other imaging methods or task‐based fMRI paradigms (Alaçam et al. 2023; Arbabshirani et al. 2013; Duda et al. 2023; Iraji et al. 2023; Lee, Smyser, and Shimony 2013). rs‐fMRI shows robustness in clinical applications, even at short scan times (2–5 min) (Duda et al. 2023) and allows identification of individual functional brain connectivity profiles (Finn et al. 2015). These features are important for clinical populations who may struggle to perform standardized tasks in the scanner.

The traditional approach to functional brain connectivity assumes a static connectivity pattern throughout the data acquisition period (Hutchison et al. 2013). However, it has been shown that spontaneous BOLD signals recorded during periods of rest display inherent spatiotemporal dynamic organization (Chang and Glover 2010; Hutchison, Womelsdorf, Gati, Everling, and Menon, 2013; Sakoğlu et al. 2010). Dynamic functional network connectivity (dFNC) is one of the strategies proposed to characterize time‐varying brain properties (Sakoğlu et al. 2010). Within this framework, the brain is partitioned into independent networks using group independent component analysis (ICA), each component with its unique temporal profile (Calhoun and Adalı 2012; Calhoun et al. 2014). The subsequent examination of time‐varying changes among component time courses, known as functional network connectivity (FNC), involves calculating cross‐correlations between brain networks (components) over time (Calhoun et al. 2014; Jafri et al. 2008). Temporal correlation patterns evolve over time, reflecting fluctuations in neural activity at the macroscopic level and provide insights into how brain networks evolve and interact over different time scales. Afterward, clustering analysis is executed on the time series of correlation patterns to identify matrices representing connectivity “states.” These states are fundamental to cognition and behavior and potentially useful for characterizing distinct clinical conditions (Calhoun et al. 2014; Hutchison et al. 2013), at least at a group level. Although patterns of both static (calculated over an entire scan) and dynamic functional connectivity exhibit sensitivity to individual variations in health and disease, dFNC is considered to be a more sensitive biomarker compared with static FNC (Damaraju et al. 2014; Jin et al. 2017; Sakoğlu et al. 2010). Altered dFNC patterns have been observed in a wide range of neurological and psychiatric disorders compared to control groups (Alaçam et al. 2023; Allen et al. 2014; Damaraju et al. 2014; de Lacy and Calhoun 2019; de Lacy et al. 2017; Duda et al. 2023; Jin et al. 2017; Lurie et al. 2020; Miller, Vergara, Keator, and Calhoun, 2016; Sakoğlu et al. 2010).

Schizophrenia (SZ), a mental disorder affecting around 1% of the world's population, and defined by cross‐sectional positive, negative, and cognitive symptoms plus longitudinal course, which results in impaired functioning (Bhugra 2005; Wyatt et al. 1995). Ongoing research aims to elucidate its currently unknown underlying mechanisms. One particular focus is on comprehending changes in dFNC, which may offer insight into dynamic brain processes associated with SZ. SZ is likely characterized by dysconnectivity, which refers to an abnormal functional integration of brain processes and implies disrupted communication between different brain regions. Individuals diagnosed with schizophrenia, particularly those with prominent hallucinations, exhibit notable decreased dynamic activity of time‐varying whole‐brain network connectivity patterns (Miller, Vergara, Keator, and Calhoun, 2016; Miller et al. 2016). Furthermore, SZ affects the sensitivity of inter‐network connectivity to broader functional brain interactions (Miller, Vergara, Keator, and Calhoun, 2016).

In healthy subjects, patterns of connectivity within the inter‐auditory–visual‐sensorimotor networks (AVSN) respond to variations in network relationships across various domains. Conversely, individuals with SZ exhibit isolated inter‐AVSN connectivity, which does not influence nor responds to changes in network relationships within domain pairs containing at least one non‐AVSN functional domain (Miller, Vergara, Keator, and Calhoun, 2016). The neural mechanisms of SZ dysconnectivity are unclear, and research continues to investigate their dynamics and clinical significance. As SZ presents as a complex disorder (or disorders) with disrupted brain network interactions at both static and dynamic levels, sophisticated approaches are needed to reveal its underlying neural mechanisms.

In recent years, there has been an increase in empirical studies focused on integrating structural and functional connectivity analyses with information theory. This combination offers a framework for advancing our understanding of brain organization (Poza, García, and Gomez‐Pilar 2021). Metrics originating from information theory, particularly those linked with entropy, have shown their ability to extract meaningful information from underlying brain networks, in both healthy and mental disorder states (Poza, García, and Gomez‐Pilar 2021). A recent study (Blair, Miller, and Calhoun 2024) investigated trajectories of transitions between dFNC states in dFNC state space by evaluating entropy production along each dimension of the proposed basis space. The authors reported that SZ patients demonstrated lower entropy, suggesting simpler trajectories of the transitions between functional brain states compared to healthy controls.

In this work, we introduced and tested a novel method that combines FNC and information theory approaches—inter‐network connectivity entropy (ICE), entropy of distribution of time‐varying connectivity strength across functional brain regions. We investigated static and dynamic ICE across 53 functional intrinsic brain networks extracted from the rs‐fMRI data of 311 subjects, comprising 151 SZ patients and 160 HC, to discern potential differences in ICE between SZ patients and controls. We aimed to determine functional brain networks that exhibit those differences and evaluate whether they manifested as higher or lower values in SZ patients relative to controls. Of note, ICE measures the randomness in the distribution of connectivity strength for a given network. Higher ICE indicates higher randomness and more heterogeneity of connectivity strength across networks to which given network is connected. In contrast, lower ICE reflects less randomness and more concentration (less heterogeneity) in the distribution of connectivity strength from given source network. In addition, we performed C‐means fuzzy clustering on functional ICE correlation matrices to uncover differences in functional entropy correlation between and within intrinsic brain networks in both the SZ and control groups. Furthermore, we employed k‐means clustering of time‐indexed ICE vectors to identify characteristic ICE states and their occupancies for each group. Our approach provides new insights into the neural mechanisms of dysconnectivity in SZ and potentially aids in developing novel biomarkers for mental disorders.

## 2 Materials and Methods

### 2.1 fMRI Data

**FIGURE 1:**
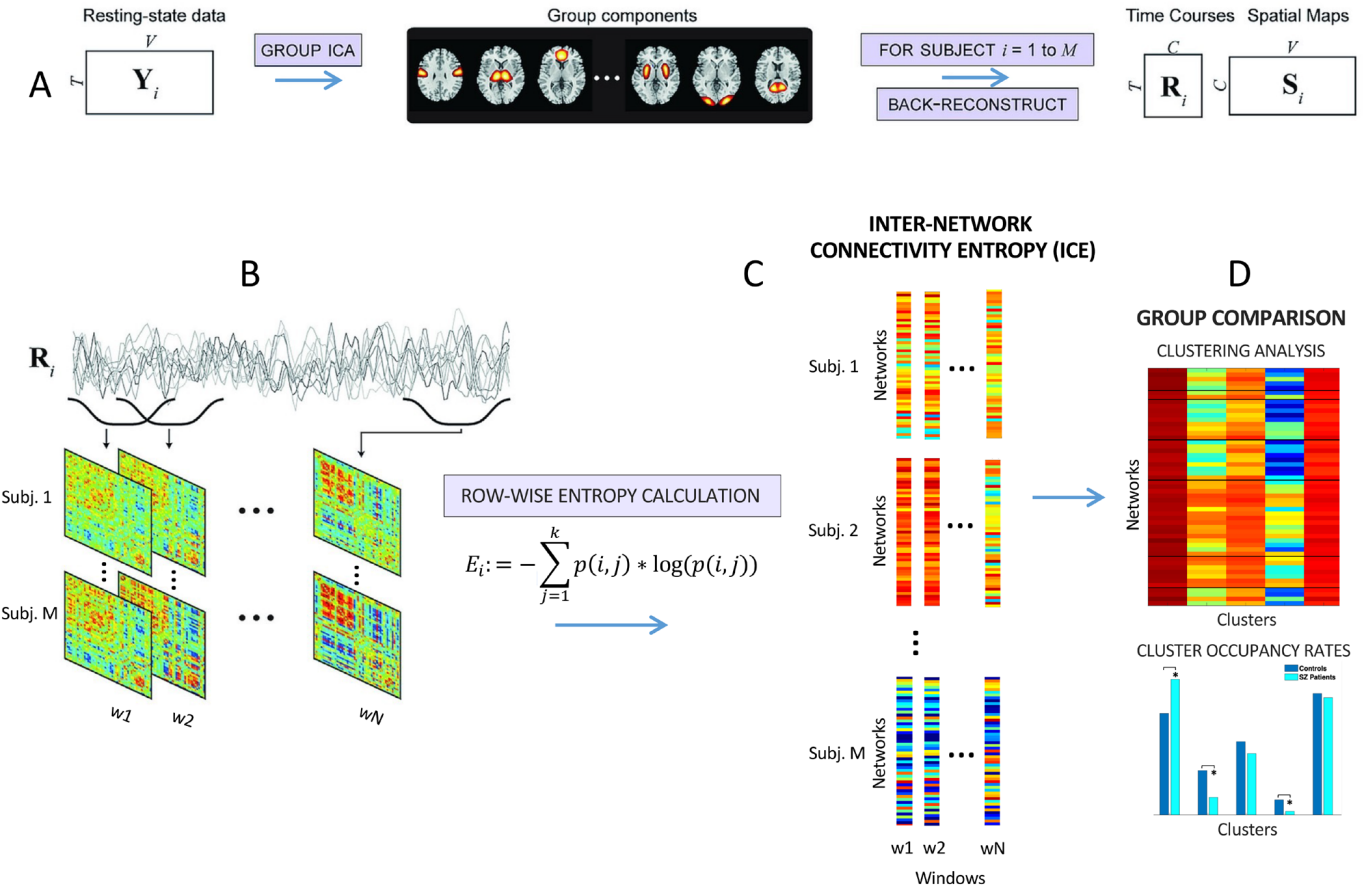
Schematics of main steps of analysis modified from Miller et al. (2016). (A) Decomposition of resting‐state fMRI data with group ICA into network spatial maps and corresponding time courses. (B) Sliding window dynamic functional connectivity is computed across all subjects. (C) Computation of inter‐network connectivity entropy (ICE) from dynamic functional connectivity matrices. (D) Clustering and regression analysis to examine group differences between SZ and HC.

We used resting‐state fMRI data collected from a total of 311 participants, comprising 160 HC and 151 individuals with SZ, matched for age and gender. The data were acquired as part of the multi‐site fBIRN project (Potkin and Ford 2009). Participants were directed to keep their eyes closed throughout the scans. Data collection occurred every 2 s (TR) for a total of 160 TRs, equivalent to 5.33 min. The data underwent preprocessing using a standard pipeline, as detailed in Damaraju et al. (2014) and Du et al. (2020) and underwent decomposition with group‐independent component analysis. This process yielded 100 group‐level functional network spatial maps along with their corresponding timecourses (Figure 1). Among these components, 53 were identified as functional brain networks, aka intrinsic connectivity networks (ICNs), in accordance with the methods described in earlier publications (Damaraju et al. 2014; Du et al. 2020). Subject‐specific spatial maps and temporal profiles were obtained using spatiotemporal regression. The temporal profiles of each subject's ICNs were detrended, orthogonally aligned with motion parameters, and despiked. Detailed description of data collection, estimation of the functional networks, their functional connectivity, and number of temporally independent sources are provided in Blair, Miller, and Calhoun (2024) and Du et al. (2020).

### 2.2 ICE

First, we calculated network connectivity distributions from dFNC matrices. Next, we determined the entropies of these distributions (inter‐network connectivity entropies [ICE]). Network connectivity distributions and ICE were calculated in static and dynamic ways, obtaining ICE aggregated over all time windows, static ICE (SICE), and window‐wise ICE or dynamic ICE (DICE).

For each functional intrinsic network i, we looked at its connectivities c(i,j) obtained from dFNC matrices across j≠i as a distribution of connectivity strengths across functional intrinsic brain networks. We mapped each $c\left(i,j\right)$ to a non‐negative translate denoted as $c^{\prime }\left(i,j\right)=c\left(i,j\right)-C_{\min}$, where $C_{\min}$ is a minimal connectivity on a global level. This translation results in positive values, allowing us to conceptualize these connectivities probabilistically. Next, we computed a probability distribution for each network *i,* $P_{i}$, consisting of the sequence of probabilities $\left\{p\left(i,1\right),p\left(i,2\right),\ldots p\left(i,k\right)\right\}$, j≠i, where $p\left(i,j\right)=\frac{c^{\prime }\left(i,j\right)}{\sum_{j=1}^{k}c^{\prime }\left(i,k\right)}$, j≠i, is a summed connectivity of network to each other network rescaled to be a distribution, k is a number of networks. Finally, we computed the connectivity entropy of this probability distribution for every network i as: $E_{i}≔-\sum_{j=1}^{k}p\left(i,j\right)\times \log\left(p\left(i,j\right)\right),\;i\in \{1,2,3,...k\},j\neq i$.

We obtained tensors of dynamic and static ICE values in dimensions of 53 × 137 × 311 and 53 × 311, respectively. Here, 53 represents the number of functional intrinsic networks, 137 indicates the number of time windows, and 311 signifies the number of subjects. Functional entropy correlation matrices of dimensions 53 × 53 for both SZ patients and controls were generated by autocorrelation of the 53 × 137 matrices of DICE for each subject. All computations and data analyses were performed utilizing custom MATLAB scripts. Connectograms depicting functional ICE correlations were generated using GIFT toolbox function “icatb_plot_connectogram” (http://trendscenter.org/software/gift) (Iraji et al. 2021) and Neuromark fMRI 1.0 template (Du et al. 2020). The implementation of our ICE approach has been incorporated into the GIFT toolbox http://trendscenter.org/software/gift.

### 2.3 Clustering Analysis

The C‐means fuzzy clustering was performed on functional ICE correlation matrices of all subjects with the Euclidean distance, 500 iterates, fuzziness parameter equal 1.05. The set of functional ICE correlation matrices was segmented into five clusters, with their centroids serving as basis correlation patterns. Each of these clusters represents distinct functional entropy correlation patterns. In C‐means fuzzy clustering, functional entropy correlation matrix for each subject is assigned a degree of membership to multiple clusters rather than a hard assignment to just one cluster. Cluster occupancy weights were derived from the fuzzy partition matrix, which contains the percentage of cluster membership for each observation. The occupancy weights, or cluster membership percentage, indicate the extent to which the functional entropy correlation pattern belongs to a specific cluster. The soft assignment provides more nuanced information about the transitions between functional ICE patterns across brain networks compared to hard clustering methods, which can capture mixed or intermediate ICE patterns. High cluster membership percentages for given cluster suggest that the corresponding functional ICE connectivity pattern is predominant, while lower percentages indicate that the functional ICE connectivity pattern is less frequently represented. Thus, occupancy weight quantifies how strongly a particular functional entropy correlation pattern is represented in the overall brain dynamics.

The k‐means clustering algorithm was applied to the time‐indexed entropy vectors partitioning data into five different clusters using Euclidean distance, 500 iterates, and 50 replicates followed by assessment of subject‐level cluster occupancy rates and dwell time for both SZ patients and HC. Number of clusters was established using the elbow criterion. We used MATLAB's functions for both k‐means and C‐means clustering.

### 2.4 Statistics

A linear regression model and two‐sample *t*‐test were employed to evaluate the relationship between SZ and ICE. The reported *p* values were adjusted for multiple comparisons using false discovery rate (FDR) at $\alpha_{\mathrm{FDR}}=0.05$. The regression model incorporated potential confounding variables such as age, gender, and mean frame displacement (motion). The diagnosis variable is binary, where “1” represents SZ and “0” represents HC. Therefore, a positive regression coefficient for diagnosis indicates a positive correlation with SZ, while a negative value of regression coefficient for diagnosis indicates a negative correlation with SZ. The Positive and Negative Syndrome Scale was used to evaluate the relationship between SZ symptom severity and ICE values.

## 3 Results

### 3.1 SZ Patients Tend to Display Higher Static and Dynamic ICE Across the Majority of Intrinsic Brain Connectivity Networks When Contrasted With Healthy Controls

**FIGURE 2:**
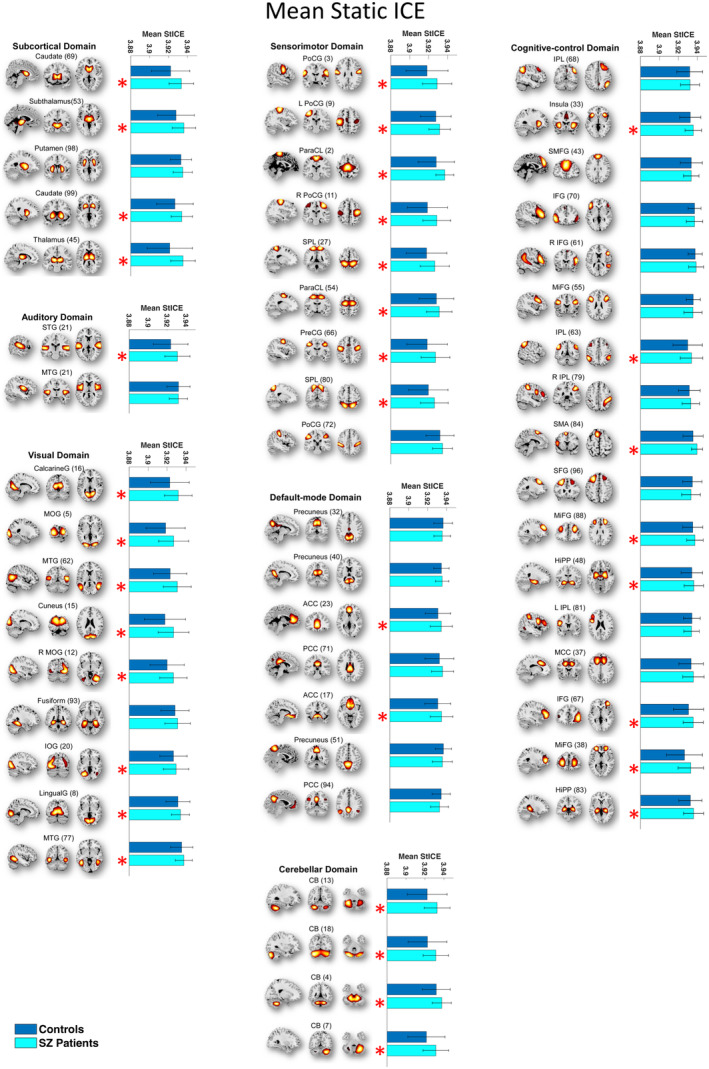
The majority functional brain networks demonstrated significantly higher mean static ICE (SICE) in SZ patients compared to control group. Networks that have significant differences in mean SICE between SZ patients and controls are shown with red “*” marks. The statistical results were acquired from the diagnosis term in univariate multiple regression models.

**FIGURE 3:**
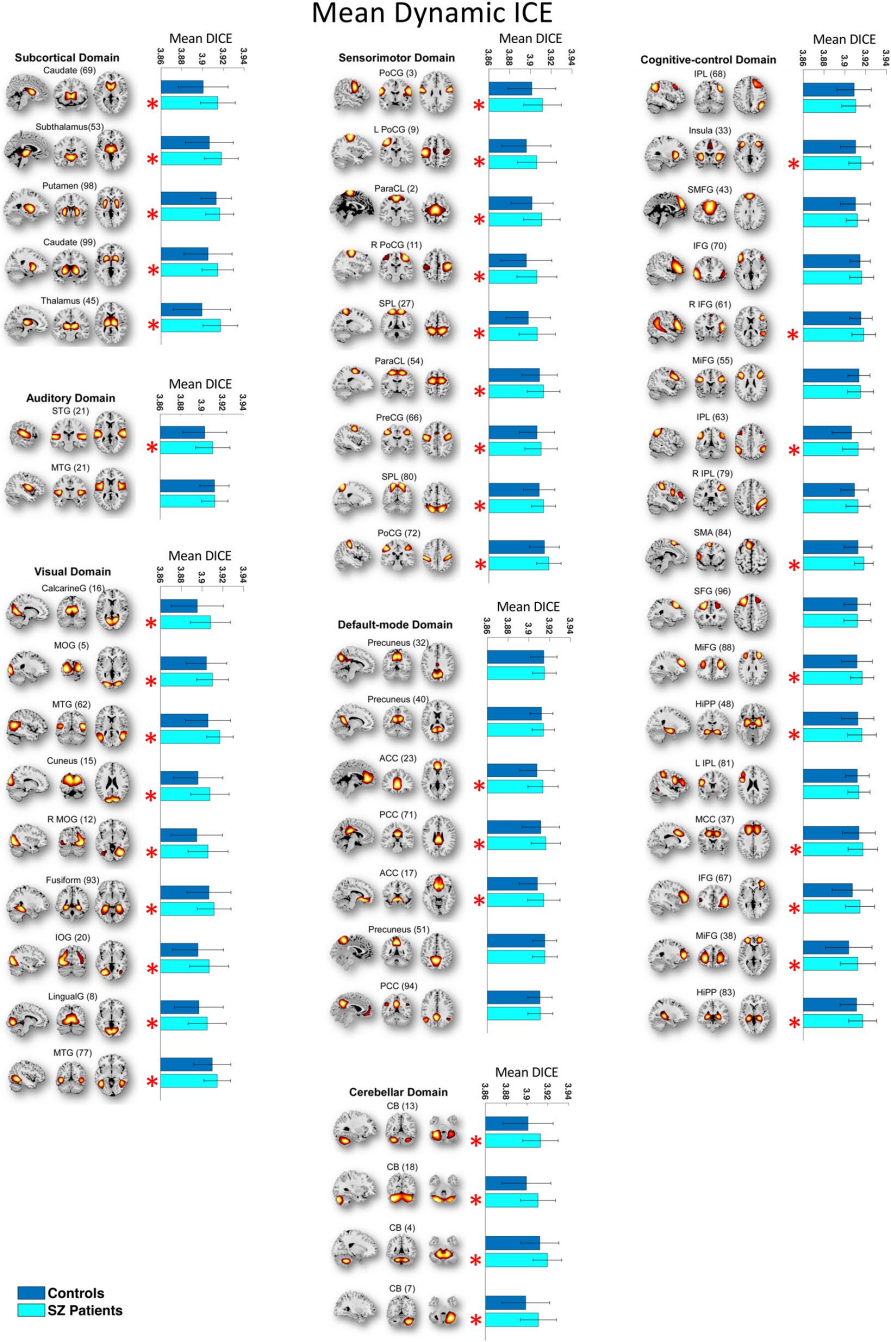
The majority of functional brain networks demonstrate significantly higher mean dynamic ICE (DICE) in SZ patients compared to control group. Networks that have significant differences in mean DICE between SZ patients and controls are shown with red “*” marks. The statistical results were acquired from the diagnosis term in univariate multiple regression models.

In our study, our goal was to examine heterogeneity in connectivity strength distributions across intrinsic connectivity brain networks in both SZ patients and healthy controls. To accomplish this, we computed the entropy of connectivity strength distributions within functional brain regions of each source network, termed as ICE. Among the 53 functional brain networks examined, 36 exhibited statistically significant difference in static ICE between SZ and control subjects (*p* ≤ 0.0274 [FDR]) (Table 1). These implicated networks encompass diverse functional brain domains, including subcortical (SC), auditory (AUD), visual (VIS), sensorimotor (SM), cognitive control (CC), default mode (DMN), and cerebellar (CB) networks. Furthermore, dynamic ICE showed even more extensive differences with 41 out of the 53 functional networks (*p* ≤ 0.0379 [FDR]) showing significant differences across the same functional brain domains (Table 1). Mean static and mean dynamic ICEs computed across 53 ICNs for both HC and SZ patients are illustrated in Figures 2 and 3.

**TABLE 1:** Mean ICE associated with intrinsic connectivity networks.

#	ICNs	SICE	DICE	#	ICNs	SICE	DICE
	Subcortical (SC)				Cognitive control (CC)		
1	Caudate (69)	+	+	26	Inferior parietal lobule (68)		
2	Subthalamus/hypothalamus (53)	+	+	27	Insula (33)	+	+
3	Putamen (98)		+	28	Superior medial frontal gyrus (43)		
4	Caudate (99)	+	+	29	Inferior frontal gyrus (70)		
5	Thalamus (45)	+	+	30	Right inferior frontal gyrus (61)		+
	Auditory (AUD)			31	Middle frontal gyrus (55)		
6	Superior temporal gyrus (21)	+	+	32	Inferior parietal lobule (63)	+	+
7	Middle temporal gyrus (56)			33	Left inferior parietal lobule (79)		
	Sensorimotor (SM)			34	Supplementary motor area (84)	+	+
8	Postcentral gyrus (3)	+	+	35	Superior frontal gyrus (96)		
9	Left postcentral gyrus (9)	+	+	36	Middle frontal gyrus (88)	+	+
10	Paracentral lobule (2)	+	+	37	Hippocampus (48)	+	+
11	Right postcentral gyrus (11)	+	+	38	Left inferior parietal lobule (81)		
12	Superior parietal lobule (27)	+	+	39	Middle cingulate cortex (37)		+
13	Paracentral lobule (54)	+	+	40	Inferior frontal gyrus (67)	+	+
14	Precentral gyrus (66)	+	+	41	Middle frontal gyrus (38)	+	+
15	Superior parietal lobule (80)	+	+	42	Hippocampus (83)	+	+
16	Postcentral gyrus (72)		+		Default mode (DMN)		
	Visual (VIS)			43	Precuneus (32)		
17	Calcarine gyrus (16)	+	+	44	Precuneus (40)		
18	Middle occipital gyrus (5)	+	+	45	Anterior cingulate cortex (23)	+	+
19	Middle temporal gyrus (62)	+	+	46	Posterior cingulate cortex (71)		+
20	Cuneus (15)	+	+	47	Anterior cingulate cortex (17)	+	+
21	Right middle occipital gyrus (12)	+	+	48	Precuneus (51)		
22	Fusiform gyrus (93)		+	49	Posterior cingulate cortex (94)	+	
23	Inferior occipital gyrus (20)	+	+		Cerebellum (CB)		
24	Lingual gyrus (8)	+	+	50	Cerebellum (13)	+	+
25	Middle temporal gyrus (77)	+	+	51	Cerebellum (18)	+	+
				52	Cerebellum (4)	+	+
				53	Cerebellum (7)	+	+

*Note:* The majority of functionally relevant intrinsic connectivity networks have significant differences in static and mean dynamic inter‐network connectivity entropies (SICE and DICE correspondingly) between SZ patients and controls. (These networks are shown with “+” marks). Statistics are obtained via linear regression to assess the impact of diagnosis on ICE, FDR < 0.05. Regression coefficients and *p* values for every observation are presented in Table S1. Numbers in brackets indicate Brodmann areas.

Next, we assessed whether SZ patients exhibited higher or lower levels of ICE compared to healthy controls. Except for the posterior cingulate cortex (ICN 49), all networks with significant differences in dynamic ICE between patients and controls demonstrated higher ICE in SZ compared to controls. While the posterior cingulate cortex network demonstrated higher static ICE in HC, no statistically significant difference in dynamic ICE was observed between SZ patients and HC in this network.

To assess the effects of age and gender on ICE, we employed a linear regression model while correcting for multiple comparisons. Our analysis indicated that gender did not significantly affect heterogeneity of inter‐network connectivity strength distribution for both static and dynamic measures, whereas age had statistically significant effect on precuneus intrinsic connectivity network for static ICE measure. In addition, we investigated the effect of the composite cognitive score and the combined effect of the composite cognitive score and diagnosis (composite cognitive score by diagnosis interaction) on ICE group differences. To this end, we added terms for the composite cognitive score and the composite cognitive score by diagnosis interaction to the regression model. The regression analysis showed that there was no statistically significant effect of either the composite cognitive score or the interaction of the composite cognitive score and diagnosis on both static and dynamic ICE.

### 3.2 Altered ICE in the Insula, Posterior Cingulate Cortex and Superior Parietal Lobule Is Associated With Severity of SZ Symptoms

Next, we investigated the relationship between static and averaged dynamic ICE values and severity of various symptoms in SZ individuals. To achieve this, we conducted a robust regression analysis, using the ICE values from 53 networks in all SZ subjects as the dependent variable. The independent variables included age, gender, motion, and 30 symptoms. By including all the symptoms in the regression model and adjusting *p* values for multiple comparisons, we identified significant symptoms that correlated with ICE values. This approach provides a more comprehensive analysis of the relationship between specific symptoms and brain network dynamics in SZ. For the static measure, results showed significant associations of increased ICE in insula (ICN 27) with disrupted attention (adjusted *p* value: 0.001177, FDR corrected); decreased ICE in posterior cingulate cortex (ICN 49) and hostility (adjusted *p* value: 0.034053, FDR corrected). For averaged dynamic measure, the significant relationship was between increased average dynamic ICE in insula (ICN 27) and disrupted attention (adjusted *p* value: 0.013863, FDR corrected), guilt feeling (adjusted *p* value: 0.023511, FDR corrected), and volition (adjusted *p* value: 0.021045, FDR corrected). Also, we found significant association of increased average dynamic ICE in superior parietal (ICN 15) lobule with guilt feelings (adjusted *p* value: 0.030028, FDR corrected).

### 3.3 SZ and Control Groups Have Distinct Distributions of ICE Across a Variety of Intrinsic Connectivity Networks

**FIGURE 4:**
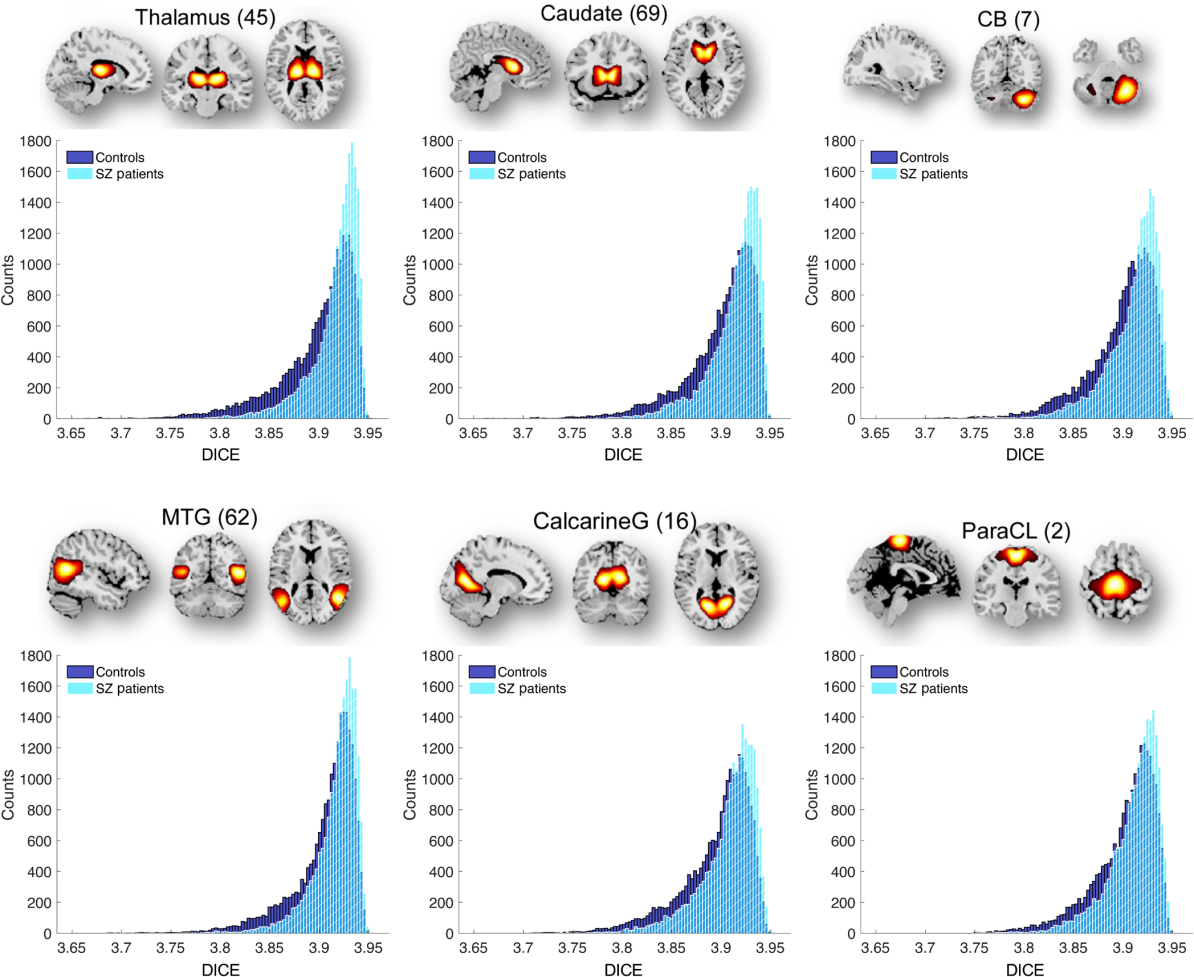
The DICE histograms characterizing SZ patients are skewed toward higher connectivity entropies and contain a larger portion of the mass at the higher end compared to the control group. Six representative functional brain networks—Thalamus (SC), Caudate (SC), Cerebellum 4 (CB), Calcarine gyrus (VIS), Middle temporal gyrus (VIS) and Paracentral lobule (SM)—show significant differences in dynamic ICE with corresponding *p* values: 2.56 × 10^−10^, 1.27 × 10^−8^, 2.98 × 10^−8^, 1.29 × 10^−7^, 2.28 × 10^−6^, 4.90 × 10^−6^. Distributions were obtained for DICE aggregated over all windows and subjects of each group.

**FIGURE 5:**
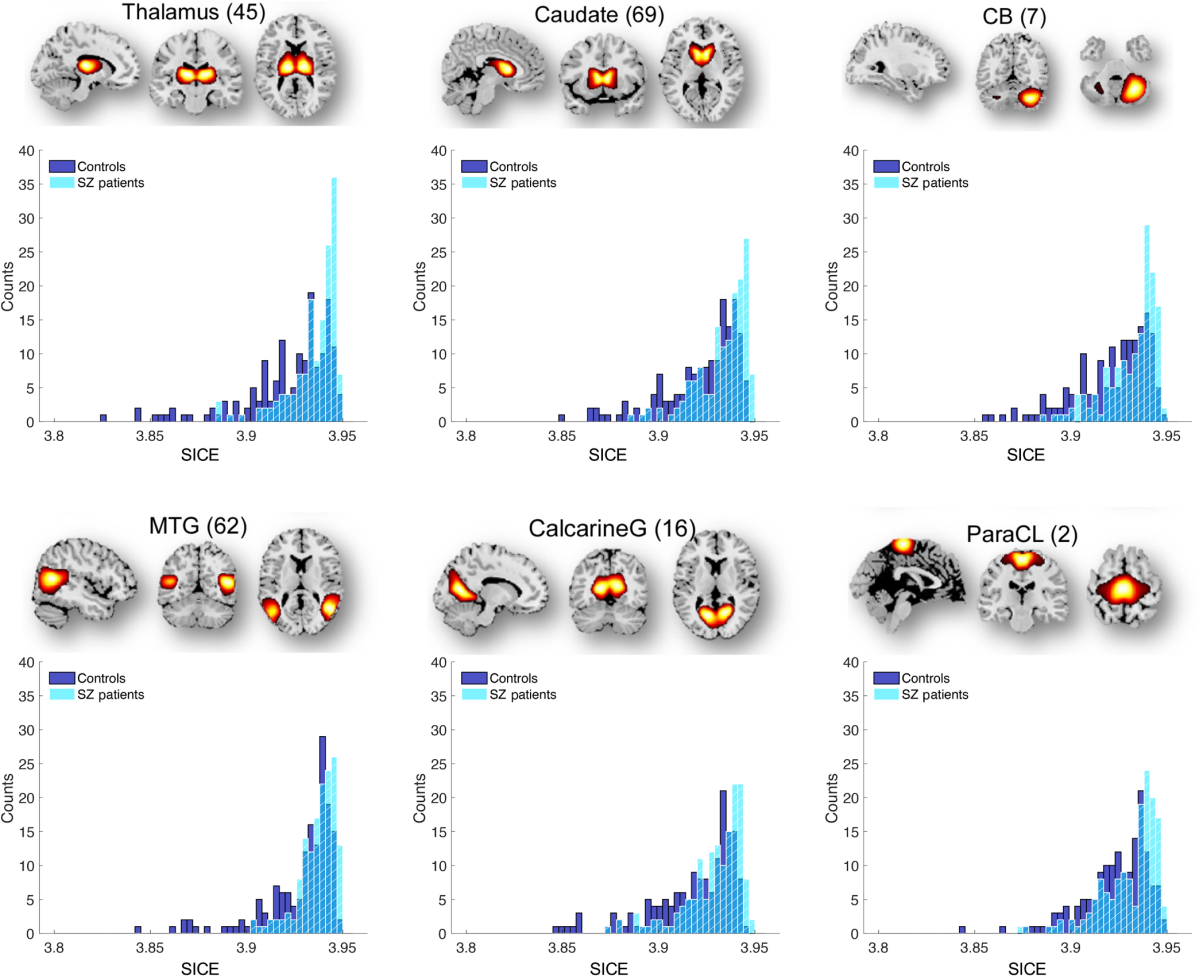
Similar to DICE histograms, SICE histograms characterizing SZ patients are skewed toward higher connectivity entropies and contain a larger portion of the mass at the higher end compared to the control group. Six representative functional brain networks—Thalamus (SC), Caudate (SC), Cerebellum 4 (CB), Calcarine gyrus (VIS), Middle temporal gyrus (VIS) and Paracentral lobule (SM)—show significant differences in static ICE with corresponding *p* values: 4.22 × 10^−9^, 3.96 × 10^−8^, 6.77 × 10^−8^, 1.66 × 10^−6^, 2.61 × 10^−6^, 8.16 × 10^−6^. Distributions were obtained averaging ICE over time windows for every subject of each group.

Mean values of dynamic ICE computed across windows and subjects provide limited information. Therefore, we examined the distributions of dynamic ICE across different subjects for all networks with statistically significant difference between patients with SZ and healthy controls. Six representative histograms of dynamic and static ICE for control and SZ groups are shown in Figures 4 and 5. The histograms are left‐skewed for both patients with SZ and controls, whereas SZ histograms have bulk of the mass at the higher end in the distributions compared to controls. Among all 41 networks with *p* ≤ 0.0379 (FDR), the distributions associated with SZ patients were shifted toward higher connectivity entropies compared to controls. This result is consistent and complementary with the findings presented in the previous section, which described a higher mean ICE in SZ patients.

### 3.4 SZ Patients Have Lower Variability in Inter‐Network Connectivity Strength Distribution Over Time Compared to the Control Group

**FIGURE 6:**
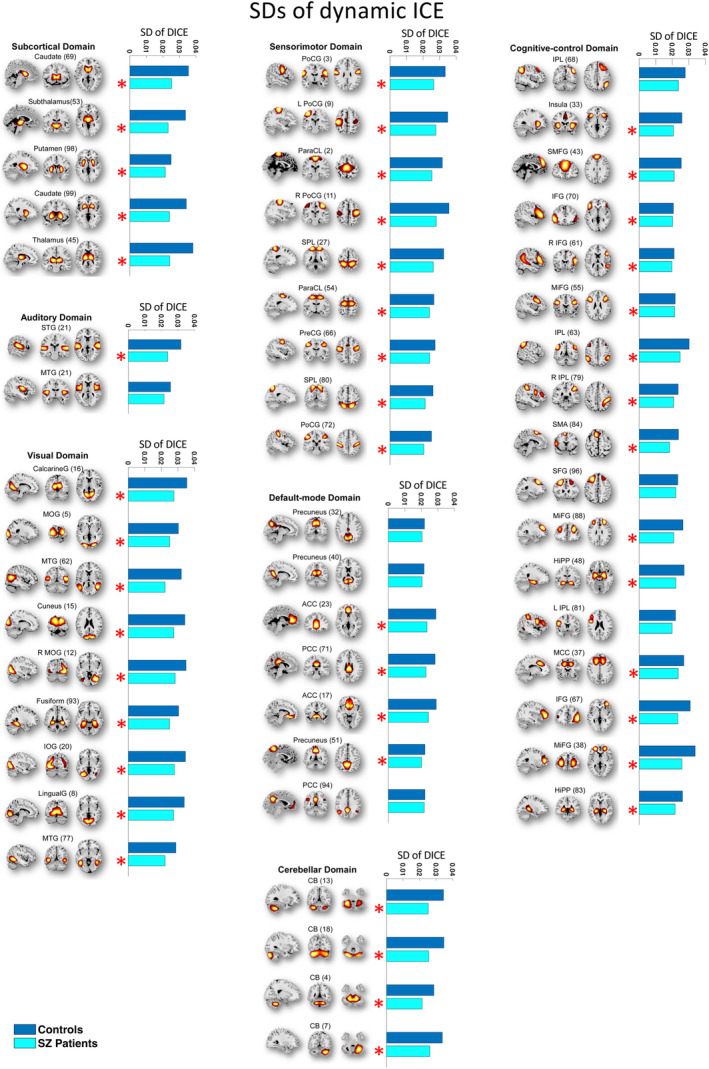
Forty‐six out of 53 functionally relevant intrinsic connectivity networks exhibit significantly lower SDs of DICE in SZ patients compared to healthy controls. These networks are shown with “*” marks. Standard deviations were calculated for seven brain domains comprising of 53 functional networks. The majority of these 46 networks have significant differences in SICE and DICE between SZ patients and controls.

To explore the variability in network connectivity strength distribution over time, we examined the standard deviations (SDs) of the dynamic ICEs across all intrinsic functional brain networks. Forty‐six of 53 functionally relevant intrinsic connectivity networks (shown with red “*” marks in Figure 6) have significantly higher SD of the DICE in HC compared to SZ patients. All functional networks with significant differences in SICE and DICE between SZ patients and controls, except posterior cingulate cortex, characterized with high variability in network connectivity strength distribution over time. Moreover, inter‐network connectivity in SZ patients exhibited a more uniform distribution, showing relatively consistent temporal patterns, rather than displaying high average entropy driven by specific periods of elevated entropy that skew the average upwards.

### 3.5 SZ Patients Have Distinct ICE Patterns in SC, AUD, SM, VIS and CB Brain Domains Compared to Healthy Controls

**FIGURE 7:**
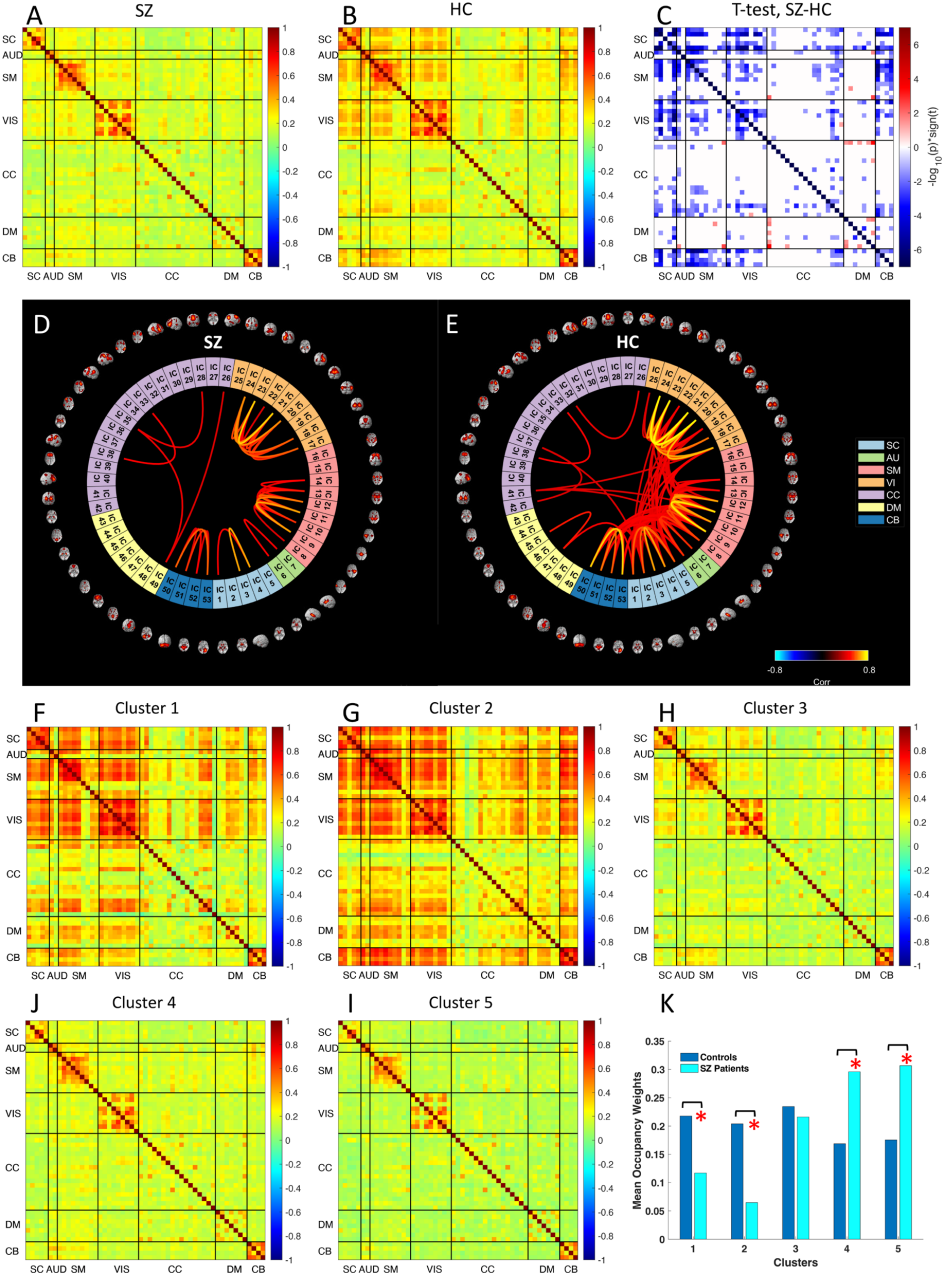
SZ patients exhibit reduced correlation of ICE between brain regions compared to controls. (A and B) Mean functional entropy correlation matrices obtained from dynamic ICE for SZ patients and control group respectively. (C) The group difference (SZ–HC) in functional entropy correlation matrices. Values are plotted as −log10(*p* value) *sign(*t* value), where statistics are obtained via *t*‐test across diagnosis groups, FDR < 0.05. The graph displays only the *p* values that correspond to statistical significance. (D and E) illustrate connectograms derived from mean functional entropy correlation matrices for both the SZ and control groups. Connections with correlation values lower than 0.4 are omitted on the connectograms. The k‐means algorithm is utilized to cluster functional entropy correlation matrices obtained for all subjects, resulting in the identification of five cluster centroids (F–I). (K) Occupancy rates across five clusters for SZ and healthy control groups.

To explore correlation of ICE between different brain domains and to find potential difference in ICE patterns between SZ and HC, we investigated whole‐brain subject‐level functional entropy correlation matrices obtained on time courses of ICE for each component. Averaged functional ICE correlation matrices for both patients with SZ and controls and their difference are presented in Figure 7A,B correspondingly. Notably that all network show either positive or no correlation in ICE for both groups. Significant differences were observed in SC, AUD, SM, VIS, and CB domains, where SZ patients have reduced correlation of ICE between and within networks of these domains, when compared with HC (Figure 7C), which is also depicted on connectograms (Figure 7D,E).

Next, we performed clustering analysis of these functional entropy correlation patterns using a C‐means clustering approach (Figure 7F–I). We thus obtained two clusters with strong, large‐scale functional entropy correlation, two clusters with weak, low‐scale correlation, and one cluster with medium functional entropy correlation. Clusters with strong, large‐scale functional entropy correlation had larger cluster occupancy weights for controls, whereas clusters with low‐scale functional entropy correlation tended to be more occupied by SZ patients (Figure 7K). The results are consistent with FNC clusters for SZ and control groups (Damaraju et al. 2014).

### 3.6 SZ Patients and Controls Exhibit Different Occupancy Rates for Clusters Exhibiting Distinct Dynamic ICE Patterns

**FIGURE 8:**
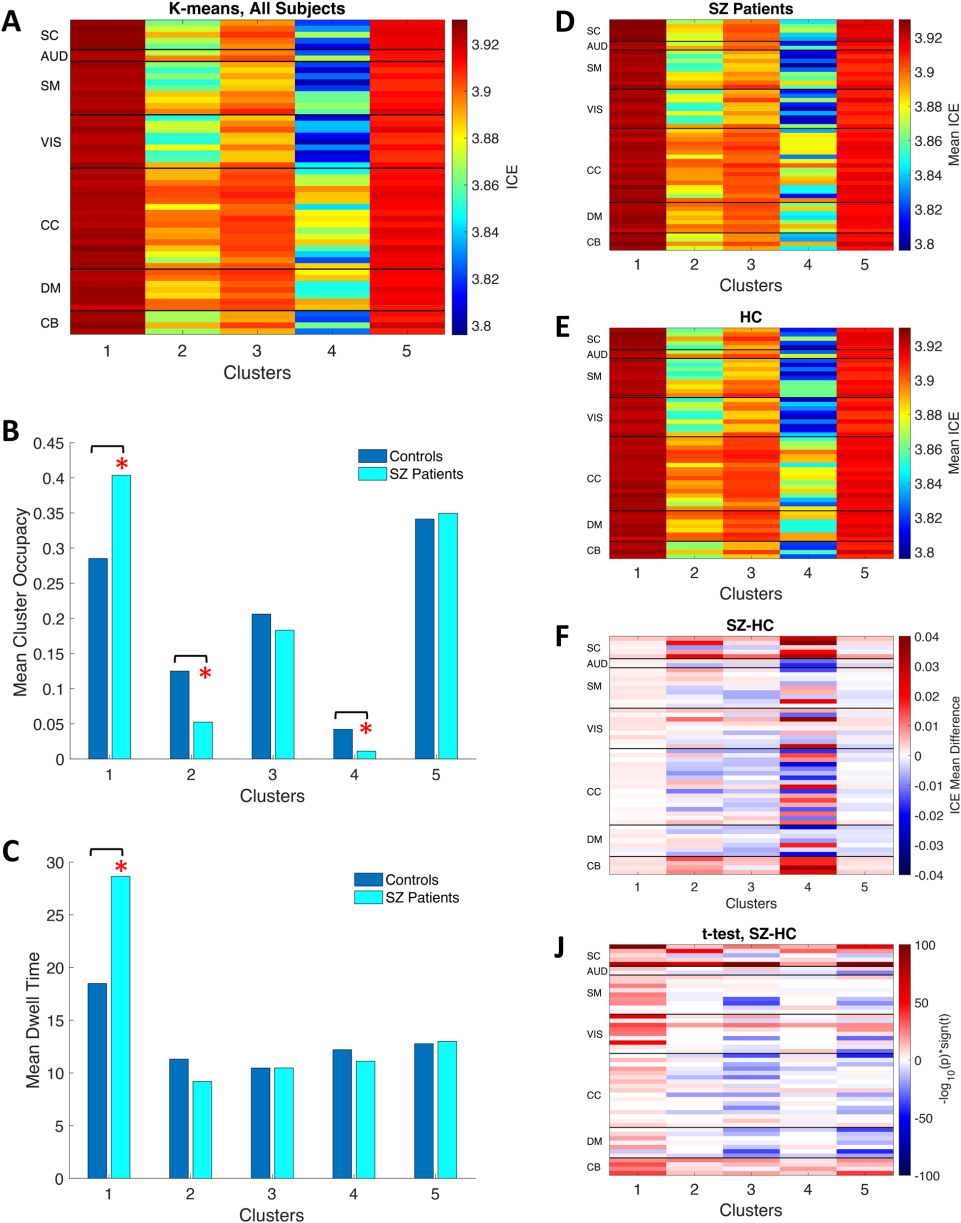
SZ patients are characterized by distinct cluster occupancy rates and different ICE patterns compared to controls (A) The k‐means clustering algorithm was applied to cluster dynamic ICE obtained for all subjects, resulting in the 5 cluster centroids. (B) Mean cluster occupancy rates for all obtained clusters. Cluster 1 with highest entropy across all networks is occupied primarily by SZ over controls, whereas cluster 4 with lowest entropy is occupied more by healthy controls. (C) Mean dwell time associated with each cluster. The mean DICE values corresponding to the five clusters are shown for SZ patients (D), HC (E) and their difference (F). (J) The group difference (SZ–HC) in mean DICE values for each ICN of each cluster. The values are plotted as −log10(*p* value) *sign(*t* value), where statistics are obtained via *t*‐test across diagnosis groups, FDR < 0.05. The graph displays only the *p* values that correspond to statistical significance.

In addition, we performed k‐means clustering on the time‐indexed ICE vectors. We obtained five cluster centroids that had different ICE patterns (Figure 8A). Two of these (1 and 5) were characterized with high entropy and another two (2 and 4) had low entropy values. Notably, clusters with high entropy exhibited high ICE across all 53 components, while clusters with low entropy displayed larger variability in ICE among functional brain networks. Interestingly, that SC, AUD, SM, VIS, and CB brain domains of clusters 2 and 4 were characterized by lower ICE values compared to other functional brain networks.

Next, we computed the mean values of subject‐level occupancy rates (Figure 8B) and dwell times (Figure 8C) for each obtained cluster. Cluster 1, with the highest entropy across all networks, exhibited significantly increased occupancy rates for SZ patients, whereas HC demonstrated significantly higher occupancy rates in clusters (2 and 4), which had low ICE values. Cluster 5 is characterized by high ICE values across all networks along with high occupancy for both groups. Mean dwell time for high‐entropy cluster 1 was highly significantly increased in SZ patients, whereas low‐entropy cluster 2 had higher mean dwell time for HCs. Despite strong effect of diagnosis on mean dwell time in clusters 2, the result was not statistically significant after FDR correction (Table S2). Clusters 3, 4, and 5 exhibited comparable mean dwell times for both SZ and HC.

We also calculated average ICE values across windows and individuals for the two groups (Figure 8D,E) and their difference (Figure 8F). Clusters 2 and 4, which exhibit low entropy, demonstrated more distinct patterns of ICE in both SZ and HC groups across various functional brain networks. The group difference (SZ–HC) in average DICE values for each cluster is shown in Figure 8J. A table with group difference *p* values (Table S3) corresponding to each ICN and each cluster is presented in the Supporting Information section. Although most ICNs in low‐entropy clusters 2 and 4 exhibited significant group differences in average DICE (Figure 8F), high‐entropy clusters 1, 3, and 5 demonstrated statistically stronger results. This phenomenon is explained by the fact that the standard deviation for ICE in most ICNs is much higher in clusters 2 and 4 than in clusters 1, 3, and 5 (Figure S1A,B). Furthermore, Cluster 4, which had the lowest ICE, exhibited a higher standard deviation of ICE for HC compared to SZ for the majority of ICNs (Figure S1C).

**FIGURE 9:**
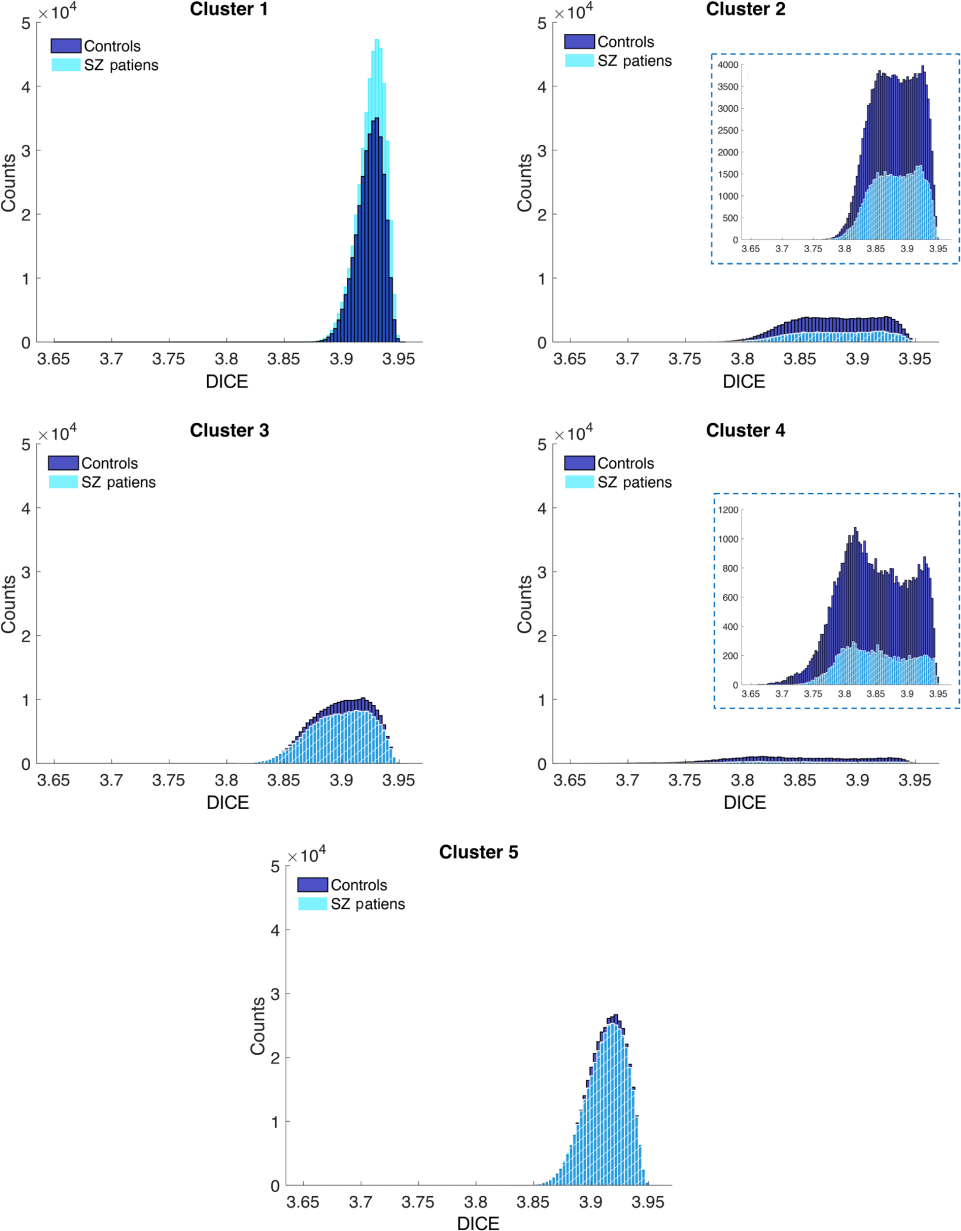
The histograms associated with the least occupied low‐entropy clusters 2 and 4 are bimodal and more broadly distributed, whereas histograms associated with high occupied high entropy clusters 1 and 5 are unimodal and more narrowly distributed. For every histogram corresponding to a specific cluster of each group, we collapsed and aggregated the 53‐length vectors present within the cluster, then showed how many of these individual elements from dynamic ICEs in this cluster are in each bin referenced on the x‐axis.

Next, we examined the distributions of dynamic ICE values for each cluster (Figure 9). The histograms validate that the patterns in the centroids are highly characteristic of the cluster elements. Histograms for less‐occupied clusters 2 and 4 exhibit a bimodal distribution and broader spread compared to the high‐occupancy, high‐entropy clusters 1 and 5, which are unimodal and narrowly distributed. Bimodality of distributions related to clusters 2 and 4 is in alignment with significantly higher SD of ICE for these clusters (Figure S1A,B). To compare SZ and HC dynamic ICE distributions, we utilized the Kolmogorov–Smirnov (K–S) test. K–S rejected the null hypothesis at 5% significance level for all clusters. This means that SZ and HC distributions of dynamic ICE associated with given cluster are statistically different for all five clusters.

## 4 Discussion

Our results demonstrated that using the proposed new measure—ICE, we were able to identify links to SZ among a range of functional brain domains: SC, AUD, VIS, SM, CC, DM, and CB. All these domains showed higher mean ICE in SZ patients compared to HC. Higher ICE associated with individuals with SZ indicates that patients demonstrate higher randomness in distribution of time‐varying connectivity strength across functional regions from each source network. This is consistent with, and extends, prior studies showing more randomness/disorganization in functional brain connectivity in SZ patients—compared to controls (He et al. 2012; Ramirez‐Mahaluf et al. 2022) as well as with (Carhart‐Harris et al. 2014) work that uses other entropy approaches not based on ICA and dFNC.

According to the brain criticality concept, healthy brains operate near a critical state, balancing between distinct dynamic states: order and chaos. This allows the brain to maintain a balance between stability and flexibility, resulting in high computational efficiency and optimized information processing (Chialvo 2010; Ma et al. 2019; O'Byrne and Jerbi 2022). It is considered that during normal waking consciousness the brain operates just below criticality and brain entropy is suppressed (Carhart‐Harris et al. 2014; Priesemann et al. 2014). Brain pathologies, including SZ, and certain pharmacological agents like psychedelics, can alter brain dynamics, potentially shifting the brain away from a balanced critical state (Alamian et al. 2022; Carhart‐Harris et al. 2014; Heiney et al. 2021). It was shown that psychedelics shift the brain dynamics toward chaos, and entropy is increased, particularly in the hippocampus and anterior cingulate cortex (Carhart‐Harris et al. 2014). These networks showed increased ICE in SZ patients in our study. This parallel is consistent with known overlaps between SZ and psychedelics effects, including some overlapping symptoms and altered brain activity, particularly desynchronization and neural activation patterns during hallucinations (Leptourgos et al. 2020; Siegel et al. 2024).

In addition, our ICE metric revealed that SZ predominantly affected intrinsic functional brain networks associated with the SC, VIS, SM, and CB, domains while approximately half of the AUD, CC, and DM ICNs were impacted (Table 1, Figure 7C). Our findings align with prior research suggesting that individuals with SZ demonstrate pervasive alterations in perception and sensory processing, distorted thinking, and impaired cognition (Kalkstein, Hurford, and Gur 2010; Uhlhaas and Singer 2010). Individuals with SZ also exhibit disruptions in the mechanisms responsible for processing AUD (Dondé et al. 2019), VIS (Adámek, Langová, and Horáček 2022; Dondé et al. 2019), and somatosensory modalities and motor functions. Also, the DMN has been widely observed to be abnormal in schizophrenia, and the mental processes hypothesized to be associated with this network are pertinent to the disease (Hu et al. 2017; Zhou et al. 2015). Abnormal activity and functional connectivity in the DMN regions of SZ patients subjects is also related to cognitive deficits and psychopathology associated with the disease (Calhoun et al. 2011; Hu et al. 2017; Zhou et al. 2007).

Reduced ICE correlation between SC, AUD, VIS, SM, and CB reflects hypoconnectivity between AUD, VIS, and SM ICNs in SZ patients reported in a prior study (Damaraju et al. 2014) and weaker connectivity between SC and CB ICNs in SZ patients in research (Soleimani et al. 2024) that used the same dataset as the current study. In addition, we revealed increased ICE correlation between CC (inferior parietal lobule) and DMN (all ICNs) in patients with SZ (Figure 7C,D). Particularly high ICE correlation was observed between inferior parietal lobule (network 26 in CC domain) and posterior cingulate cortex (network 49 in DM domain) in patients with SZ (Figure 7D).

Furthermore, we showed that SZ patients tended to have larger occupancy weights in clusters characterized by weak, low‐scale functional entropy correlation. In contrast, the control group demonstrated higher occupancy weights in clusters with strong, large‐scale functional entropy correlation. These results are consistent with, and extend, FNC state differences between SZ patients and controls reported by Damaraju (Damaraju et al. 2014). Their study demonstrated that clusters characterized by weak and low‐scale functional connectivity have greater occupancy among SZ patients compared to HC, whereas clusters with strong and large‐scale connectivity are predominantly occupied by HC rather than SZ patients.

We demonstrated that both static and dynamic mean ICE were higher in SZ patients than in healthy controls. Histograms of both static and dynamic ICE for SZ patients have a larger portion of the mass at the higher end of the distributions compared to controls. Nevertheless, dynamic ICE analysis revealed additional parameters that transiently discern SZ patients from controls. Thus, SZ subjects demonstrated less temporal variability in their network connectivity strength distributions across ICNs, maintaining relatively consistent levels of ICE compared to controls. In addition, dynamic ICE analysis enabled us to reveal that the human brain can function in distinct states characterized by different ICE patterns: states with uniformly high entropy in connectivity strength for all ICN (states 1 and 5) and states with relatively low and uneven entropy in connectivity strength across different brain networks (clusters 2 and 4) (Figure 8A). Individuals with SZ have larger occupancy rates for state 1 with the highest ICE, whereas HC have higher occupancy for low‐entropy states 2 and 4 with more structured given the network's connectivity to all the other brain networks (Figure 8B). Moreover, the high‐entropy states 1 and 5 with high entropy are largely occupied by both HC and SZ when compared with low‐entropy states 2 and 4. States with lower or mixed entropy are relatively rare and significantly rarer in SZ patients. Thus, broadly speaking, dynamic ICE analysis reveals a prevailing tendency for the brain to circulate through connectivity patterns with relatively high entropy levels, which aligns with complexity in the distribution of time‐varying connectivity strength across functional brain networks. Thus, normal brain function appears to involve predominantly less organized/structured connectivity patterns with intermittent transitions into more focused patterns. Individuals with SZ are significantly less likely to exhibit transitions to these more focused and structured connectivity patterns.

It is interesting to observe that both SZs and HCs exhibit the highest mean dwell time in high‐entropy state 1 compared to other states indicating that this state dominates brain dynamics in both groups. However, SZ patients have a much longer mean dwell time for the state 1 compared to controls, with diagnosis showing strong and highly significant effect after FDR correction. This extended persistence in high‐entropy state suggests that SZ subjects may have difficulties in transitioning out of this high‐entropy state, potentially representing a pathological feature of SZ.

It is important to notice that many intrinsic functional brain networks exhibit the most noticeable group differences in states (1, 3, and 5), where ICE is high for majority of ICNs (Figure 8J). Also, cluster 1, with the highest DICE and highest occupancy and dwell time for SZ patients is an only cluster where all ICNs (except for precuneus, ICN of DMN) have higher DICE for SZ patients than HCs. Particularly SC, SM, VIS, and CB brain domains have significantly higher DICE in SZ patients indicating that SZ patients' brain dynamics are predominantly characterized by more chaotic/less organized functional connectivity patterns. It is also crucial to observe impaired ability of SZ subjects to achieve states (clusters 2 and 4) where specific networks ‐ SC (particularly subthalamus/hypothalamus [ICN 2] and thalamus [ICN 5]), VIS (particularly middle temporal gyrus [ICN 19]), and CB networks ‐ normally concentrating their connectivity in specific brain regions in a more structural way, as seen in HCs. This defict in achieving more structured and stable connectivity patterns centered at thalamus and VIS domains aligns with previous work (Zhang et al. 2021) reporting sensorimotor‐thalamic imbalance in SZ patients.

Finally, we revealed significant associations between altered ICE and severity of specific symptoms in SZ patients, particularly for such ICNs as insula, posterior cingulate cortex and superior parietal lobule ICNs. This implies that the impaired distribution of connectivity strength from these networks across functional regions is related to severity of specific SZ symptoms. Our static and dynamic ICE measures provided complementary information that increased ICE in insula of SZ patients is related to disrupted attention, volition, and guilt feelings. Our finding are supported by previous studies documenting the well‐established role of the insula as a key integration hub across neural circuits underlying cognitive and social–emotional disturbances in SZ patients (Gebhardt and Nasrallah 2023; Kurth et al. 2010). Furthermore, our findings add to the growing evidence that insula abnormalities could serve as potential biomarkers for SZ risk (Gebhardt and Nasrallah 2023). In addition, the association between altered ICE in posterior cingulate cortex and superior parietal lobule ICNs of SZ patients and SZ symptoms is consistent with previous findings that posterior cingulate cortex as a key hub within the DMN, which plays a central role in neuropsychiatric disorders, including SZ (Hu et al. 2017; Leech and Sharp 2014). Previous research (Das et al. 2020; Yildiz, Borgwardt, and Berger 2011) highlighted parietal lobule as an essential locus of multisensory integration. Dysconnectivity in this region is linked to psychotic‐like experiences and the severity of disorganization symptoms in SZ.

### 4.1 Limitations and Future Directions

Despite offering novel insights into time‐varying heterogeneity of brain network connectivity in healthy and disease states using a novel ICE approach, several limitations warrant consideration. First, the study's generalizability may be limited by the size and characteristics of the dataset used, which included 311 participants, comprising 151 SZ patients and 160 age and gender‐matched healthy controls. Validation with larger, independent samples could enhance population representation and improve reliability of the findings. Second, this study does not account for several potential confounding factors including use of antipsychotics and other psychotropic medications, smoking, and prior substance use. Also, it would be interesting to explore the relationships between ICE findings and illness characteristics, including age of SZ onset, duration of untreated illness and total illness duration, specific cognitive deficits, and non‐psychotropic medication use. Third, functional dysconnectivity, particularly in the somatomotor network, is reported to be transdiagnostic across multiple mental health disorders, where disorder‐specific patterns were observed in various prefrontal cortex regions (Huang et al. 2020; Ma et al. 2019; Zhang et al. 2021). Therefore, it will be important to investigate ICE profiles across different psychiatric conditions to identify illness‐specific patterns of ICE across brain domains.

Fourth, recent work has suggested that functional connectivity over time can be influenced by non‐neuronal signals in the fMRI data (Korponay, Janes, and Frederick 2024). This issue should be considered as a topic of future work that includes approaches evaluating the effect of arousal during scanning and novel denoising procedures that mitigate these effects on functional connectivity.

Fifth, functional connectivity in fMRI, especially in SZ, can be impacted by anatomical distance between brain regions. Previous study (Alexander‐Bloch et al. 2013) showed that childhood‐onset SZ patients had reduced functional connectivity strength, especially for short distances, resulting in significantly increased global mean connection distance of thresholded graphs. The current analysis does not quantify the effects of anatomical distance on ICE or dwell ratios. To address this, in the future work, we will investigate whether the changes in ICE and dwell ratios are modulated by the anatomical distance between brain nodes. This could involve calculating the Euclidean distances between brain regions and examining correlations between distance and the strength of altered ICE in SZ versus healthy controls. Such an analysis could help determine whether the observed deficits are predominantly associated with long‐distance connections.

Sixth, SZ is characterized by heterogeneity in brain structure and function that can be observed across different levels. For example, there are several subgroups among SZ patients based on the patterns of cortical thickness described in a prior study (Pan et al. 2020). Because our ICE measure is based on functional network connectivity, which is indirectly related to cortical thickness, the different patterns of cortical thickness may be indirectly reflected in our observed ICE patterns. While our current approach focused on clustering, which tries to divide the data into more homogeneous groupings within an admittingly heterogenous data set, further investigation is needed to fully address the heterogeneous nature of schizophrenia.

Finally, dynamic functional connectivity abnormalities have been found in unaffected siblings of individuals with SZ, despite these siblings being free of symptoms, medications, and disability (Guo et al. 2018). This suggests that the dynamic connectivity disruptions reflected in dynamic ICE may represent both state‐dependent phenomena associated with the illness and possible markers of SZ risk.

### 4.2 Conclusion

The proposed ICE measure together with functional brain connectivity analyses provides simple and reliable way to summarize time‐varying FNC data and investigate group effects for potential clinical application. In addition to the advantages of the time‐varying whole‐brain FNC approach—such as robustness, reproducibility, and freedom from constraints related to the selection of specific seeds or regions of interest—our approach provides a new level of understanding of both physiological and pathophysiological brain states. First, both static and dynamic ICE measures showed that SZ patients exhibit greater randomness/disorganization in the distribution of connectivity strength across various intrinsic connectivity networks spanning a wide range of functional brain domains, including subcortical, auditory, VIS, sensorimotor, cognitive control, default mode, and CB regions when compared with control group. Second, in general, SZ brains are characterized by weak, low‐scale functional entropy correlation patterns across various functional brain regions, while healthy brains tend to show strong, large‐scale functional entropy correlation. The dynamic ICE measure complements and extends our findings obtained with static ICE, revealing that, first, the healthy brain primarily navigates through complex, less focused connectivity patterns, with occasional transitions into more organized configurations of a given network's connectivity to all other brain networks. In contrast, SZ patients' brains communicate through more disorganized connectivity patterns and fail to achieve more focused functional connectivity patterns, especially evident in ICNs associated with SC (particularly subthalamus/hypothalamus and thalamus), VIS (particularly middle temporal gyrus), and CB ICNs. Second, ICE of SZ patients shows significantly less variability over time compared to controls, suggesting lower temporal dynamics in functional connectivity strength distribution in SZ. Our methodology has potential applications beyond schizophrenia, offering a connectivity‐based foundation for classifying and comparing the impact of various diseases and studying the healthy brain‐behavior relationships.

## Conflicts of Interest

The authors declare no conflicts of interest.

## Supporting information


Data S1.

## Data Availability

Due to IRB restrictions, the FBIRN data analyzed in this study cannot be shared without specific licenses. However, the dataset can be accessed upon request by contacting Dr. Theo G.M. van Erp at tvanerp@hs.uci.edu, who will facilitate the interaction with the IRB.
